# Photosynthesis and chlorophyll fluorescence of Iranian licorice (*Glycyrrhiza glabra* l.) accessions under salinity stress

**DOI:** 10.3389/fpls.2022.984944

**Published:** 2022-10-07

**Authors:** Seyyed Sasan Mousavi, Akbar Karami, Filippo Maggi

**Affiliations:** ^1^ Department of Horticultural Science, School of Agriculture, Shiraz University, Shiraz, Iran; ^2^ Chemistry Interdisciplinary Project (ChIP), School of Pharmacy, University of Camerino, Camerino, Italy

**Keywords:** electrolyte leakage, leaf area, licorice, photosynthesis, relative water content, transpiration

## Abstract

While salinity is increasingly becoming a prominent concern in arable farms around the globe, various treatments can be used for the mitigation of salt stress. Here, the effective presence of *Azotobacter* sp. inoculation (A_1_) and absence of inoculation (A_0_) was evaluated on Iranian licorice plants under NaCl stress (0 and 200 mM) (S_0_ and S_1_, respectively). In this regard, 16 Iranian licorice (*Glycyrrhiza glabra* L.) accessions were evaluated for the effects on photosynthesis and chlorophyll fluorescence. Leaf samples were measured for photosynthetic pigments (via a spectrophotometer), stomatal and trichome-related features (via SEM), along with several other morphological and biochemical features. The results revealed an increase in the amount of carotenoids that was caused by bacterial inoculation, which was 28.3% higher than the non-inoculated treatment. Maximum initial fluorescence intensity (F0) (86.7) was observed in the ‘Bardsir’ accession. Meanwhile, the highest variable fluorescence (Fv), maximal fluorescence intensity (Fm), and maximum quantum yield (Fv/Fm) (0.3, 0.4, and 0.8, respectively) were observed in the ‘Eghlid’ accession. Regarding anatomical observations of the leaf structure, salinity reduced stomatal density but increased trichome density. Under the effect of bacterial inoculation, salinity stress was mitigated. With the effect of bacterial inoculation under salinity stress, stomatal length and width increased, compared to the condition of no bacterial inoculation. Minimum malondialdehyde content was observed in ‘Mahabad’ accession (17.8 μmol/g _FW_). Principle component analysis (PCA) showed that ‘Kashmar’, ‘Sepidan’, ‘Bajgah’, ‘Kermanshah’, and ‘Taft’ accessions were categorized in the same group while being characterized by better performance in the aerial parts of plants. Taken together, the present results generally indicated that selecting the best genotypes, along with exogenous applications of *Azotobacter*, can improve the outcomes of licorice cultivation for industrial purposes under harsh environments.

## 1 Introduction

As a perennial plant of the Fabaceae family, licorice (*Glycyrrhiza glabra* L.) has important constituents in its roots and leaves, thereby generating a research-based focus on food technology and pharmaceutical applications (Heidari et al., 2021). Industries that are involved in the production of beverages, candy, and cigarettes, all employ active components derived from licorice (Esmaeili et al., 2019). However, *G. glabra* is a much sought-after species that is endangered due to overharvesting and, thus, is under environmental protection in many Iranian ecosystems (Esmaeili et al., 2020). As a matter of its economic importance and risk of depauperating, there is a scientific imperative to cultivate and domesticate this plant (Esmaeili et al, 2020; Sharma et al., 2021; Haghighi and Saharkhiz, 2022). An obstacle that usually limits licorice cultivation is soil salinity which has become a serious problem for plants in expanded areas of cultivation, thereby lowering crop yields in affected parts of the world (Mukhopadhyay et al., 2021). An approximate 5.2 billion hectares of fertile lands are currently affected by high salinity. The accumulation of salt in the soil is caused by insufficient irrigation water, leaching, and drainage (Mishra et al., 2021). While sodium chloride is a major constituent of saline soils, chloride ions are considered as toxic for plants (Joshi et al., 2022). High levels of chloride ions usually cause retardation of plant growth (Joshi et al., 2022). Salinity also affects the soil structure, plant growth and crop yield (Hopmans et al., 2021). To minimize such harm, it is necessary to understand how plants react to salt stress. High salt concentrations put plants under osmotic and ionic stress which, ultimately, adversely affects photosynthesis in all stages (Gebrehiwet et al., 2021). Photosynthesis is a multi-component process that can be disturbed by stress factors at any stage, and the overall photosynthetic potential can become impaired in a plant. When plants are exposed to salinity, their stomata close and limit the extent of photosynthesis (Hnilickova et al., 2021). Increased levels of Na^+^ and Cl^-^ in leaf tissues can have a big impact on metabolic activities that can be suppressed limiting photosynthesis itself. The decrease of leaf area and leaf surface expansion is a response to salt stress given by an increase of salt concentration (Lu et al., 2021). With prolonged salt stress, leaf senescence, chlorosis and shedding will happen. Excessive amounts of salt tend to reduce the amount of pigment (chlorophyll) in the leaves, as well as the available area for photosynthetic efficiency (Razmjooei et al., 2022). NaCl decrease the rate of photosynthetic gas exchange but enhance CO_2_ assimilation, along with photosystem II [PS(II)] and photosystem I [PS(I)] activity and photochemical efficiency. During salinity stress, PSII activity is inhibited and, thus, a key component of the electron transport chain becomes adversely affected (Çiçek et al., 2018). Increased diffusional resistances will cause by salt stress may be fully overcome by exposing leaves to very low CO_2_ (Liu et al., 2021). Due to stomatal limits, salt exposure impairs photosynthesis and leads to lower carbon assimilation, decreasing chlorophyll and carotenoid content. Salt stress also alters the size and density of stomata, so that stomatal conductance decreases (Zhang et al., 2021). Parallel to a salt-induced increase in the minimal fluorescence (F0) and the noticeable drop in the maximal fluorescence (Fm), salt stress may also limit maximum quantum yield (Fv/Fm) (Oláh et al., 2021). In a light-adapted state, it could potentially result in a decrease in photochemical quenching (qP), associated with a significant increase in the coefficient of nonphotochemical quenching (Navarro-León et al., 2021; Zhang et al., 2021). As a matter of recent research, various strategies are used for mitigating the effects of salinity, such as the use of salinity-resistant plant cultivars and simultaneous use of growth-promoting bacteria (Sapre et al., 2018; Riahi et al., 2020; Yaghoubian et al., 2021). Plant-associated microbes can mediate crucial processes in the physiological function of plants, thereby assisting in tolerance to a variety of stresses (Shekhawat et al., 2022). Growth-promoting bacteria are microbes that have the ability to live in symbiosis with various plant species, including those of the Fabaceae family. One of the most common and prominent genera for this purpose is *Azotobacter* which is reported as a contributor to nitrogen availability for crop growth (Aasfar et al., 2021; Nongthombam et al., 2021). *Azotobacter* has the ability to fix atmospheric nitrogen and produce phytohormones, making it the most effective and widespread type of Plant Growth-Promoting Bacteria (PGPB) (Aasfar et al., 2021; Nongthombam et al., 2021). *Azotobacter* also produces chemicals that alter plant growth and morphology (Dar et al., 2021; El-Beltagi et al., 2022). PGPRs enhance soluble sugar, protein, proline content leading to a higher water potential gradient and improving water uptake and plant growth under stress condition. They also produce growth regulators such as auxin, cytokinin and gibberellin which are important to stimulate cell division (Shaffique et al., 2022). Other various PGPB-mediated mechanisms, whether direct or indirect, include biofilm formation, extracellular polymeric substance production, enhancement of nutrient uptake, phosphate solubilization and mineralization, siderophore production, improvement of antioxidant activities, and 1-aminocyclopropane-1-carboxylic acid (ACC-deaminase) activity (Khatami et al., 2022; Mousavi and Karami, 2022; Tran et al., 2022). Licorice is known as a tolerant plant to salinity and somehow it mentioned as a halophyte plant (Behdad et al., 2021). The aim of this study is to evaluate how different licorice accessions respond to salinity stress and how Azotobacter help these accessions to be less damaged under salinity stress; and finally, more resistant accessions will be selected. The present research also was an attempt to study how the photosynthetic features of 16 Iranian licorice accessions change under the effects of salinity stress and inoculated growth-promoting bacteria to select more tolerant accessions for cultivation.

## 2 Materials and methods

### 2.1 Experimental design, plant material, and soil preparation

**Figure 1:**
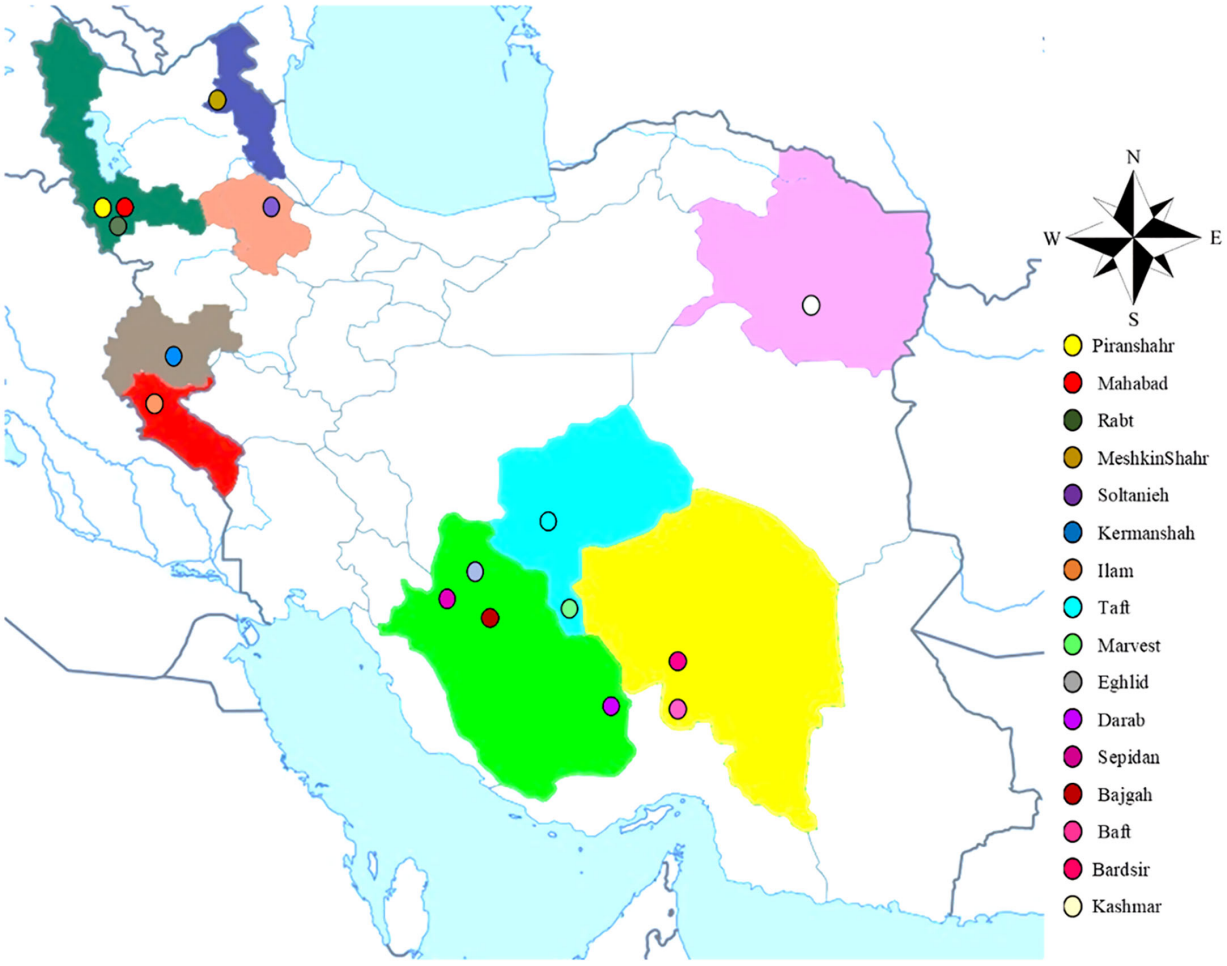
The collection areas for the investigated accessions of *G. glabra*.

The current research (2020- 2021) was carried out inside a greenhouse at the School of Agriculture, Bajgah Region, Shiraz University, Shiraz Iran, at a geographical location of 29°43’37.77” N and 52°35’12.84” E. The treatments included two levels of *Azotobacter* sp. (control: no-bacteria treatment and bacterial treatment), two levels of salinity (control: no-salt treatment and salt treatment at 200 mM NaCl), and 16 accessions of Iranian licorice plants. Thus, a total of 64 treatments were considered with three replicates, corresponding to a total of 192 pots. The sixteen licorice cultivars were gathered from various areas of Iran (**Table 1**, **Figure 1**). The collected rhizomes were cultivated in pots and placed in a greenhouse for adaptation (16 h of light, 25-28°C, 8 h in a dark environment, and 120 µmol m^-2^ s^-1^ photon flux density). One year later, the adapted accessions were propagated using rhizome cuttings. The rhizomes were cut into equal-sized pieces (15 cm long and 2 cm in diameter) with sharp garden shears. They were dipped in a fungicide solution (Benomyl 1%) and cultured in disinfected plastic pots (35×31 cm) with previously prepared soil (i.e., sieved field soil and sand (1:1 ratio)). The physicochemical properties of the soil were measured (**Table 2**). After one year, the rooted cuttings of all accessions were selected for inclusion in the experiment.

**Table 1:** Geographical characteristics of different *G. glabra* accessions collected from Iran.

No.	Accessions	Province	Longitude (E)	Latitude (N)	Altitude (m)	Voucher Number
1	Eghlid	Fars	52°29′37.9″	30˚44′ 30.8″	2319	MPH-2670-1
2	Bajgah	Fars	52°35′ 17.98″	29˚43′ 26.14″	1798	MPH-2670-2
3	Darab	Fars	54°25′ 37.64″	28˚43′ 3.95″	1081	MPH-2670-3
4	Sepidan	Fars	52°00′ 41.5″	30˚13′ 21.5″	2157	MPH-2670-4
5	Ilam	Ilam	46°17′ 43.72″	33˚40′ 49.64″	1032	MPH-2670-5
6	Baft	Kerman	56°27′ 57.6″	29˚15′ 7.1″	2241	MPH-2670-6
7	Bardsir	Kerman	56°15′ 21.94″	29˚52′ 40.41″	2338	MPH-2670-7
8	Kashmar	Razavi Khorasan	58°27′ 51.07″	35˚23′ 59.70″	1632	MPH-2670-8
9	Kermanshah	Kermanshah	46°59′ 21.37″	34˚23′ 05.91″	1371	MPH-2670-9
10	MeshkinShahr	Ardabil	47°42′ 54.80″	38˚25′ 01.10″	1412	MPH-2670-10
11	Taft	Yazd	53°50′ 59.3″	31˚39′ 44.1″	2286	MPH-2670-11
12	Marvest	Yazd	54°13′ 51.9″	30˚26′ 59.8″	1542	MPH-2670-12
13	Soltanieh	Zanjan	36˚24′ 40.21″	48°44′ 19.40″	1842	MPH-2670-13
14	Rabt	West Azarbaijan	36˚12′ 41.35″	45°31′ 54.68″	1075	MPH-2670-14
15	Piranshahr	West Azarbaijan	36˚37′ 42.95″	45°07′ 54.92″	1492	MPH-2670-15
16	Mahabad	West Azarbaijan	36˚48′ 12.68″	45°43′ 09.53″	1410	MPH-2670-16

**Table 2:** Physiochemical properties of the soil used in the present experiment.

Soil texture	Sand	Silt	Clay	OM^*^	N		Cu-DTPA	Mn-DTPA	Zn-DTPA	P-Olsen	K	Fe		EC	pH
			%						mg/kg					ds/m	-
Clay-loam	50	11	39	0.9	0.14		0.93	4.3	0.23	8	53	4.62		1.4	7.6	

In table *OM stands for organic matter.

### 2.2 Bacterial solution preparation and treatments application

Bacteria from the genus *Azotobacter* sp. (endemic to Iran) were cultivated on a nutrient broth medium (on a temperature-controlled shaker for 24 h at 28 ± 2°C) from a reputable bacterial collection in a private research institute in Iran. At 600 nm, the bacterial density was measured. Following a centrifuge stage (10,000 ×rpm, 5 min), the procedure foresees an inoculum (10^6^ CFU/mL) from a fresh bacterial culture of *Azotobacter* sp. The suspensions were utilized to treat licorice plants (Razmjooei et al., 2022). In the case of *Azotobacter* treatments, the bacterial solution was sprayed on the roots of cuttings until the roots became thoroughly wet. The roots in the non-bacterial treatment were sprayed with distilled water and were then cultivated in pots (25 kg) to be maintained for 11 months before applying the salinity treatments. The salt-treated plants were irrigated every five days (for two months) with a salt solution of 200 mM (to prevent osmotic shock). The salt concentration was chosen based on pre-treatments. The irrigation done based on field capacity (10%). The salt solution was added stepwise to the pots. The control plants and salt-treated plants were kept under saline stress in a greenhouse (16 h light, 25-28°C, 8 h in a dark environment, and 120 µmol m^-2^ s^-1^ photon flux density, at 15-21°C for two months). Finally, the aerial parts and roots of the plants were harvested. To measure several parameters of the fresh fully expanded leaves and roots, the plants (matured plants- two months after treatment application) were plucked out of the pots and were stored at -80°C. Some leaves and roots were dried for the measurement of other parameters.

### 2.3 Measurable parameters

In this experiment, several photosynthetic characteristics were measured, i.e., total chlorophyll content, chlorophyll b, chlorophyll a, total carotenoids, chlorophyll fluorescence, transpiration rate, photosynthesis rate, carboxylation efficiency, stomatal conductance, sub stomatal CO_2_, number of trichomes, number of stomata, stomatal length, stomatal width, leaf area, electrolyte leakage, membrane stability index (MSI), malondialdehyde (MDA), relative water content (RWC), and water use efficiency (WUE).

#### 2.3.1 Photosynthetic pigments

A fresh sample (500 mg) was collected, chopped into small pieces, and poured as a suspension in dimethyl sulphoxide (DMSO) (2 mL). The test tubes were incubated in an oven at 60° C for 12 h. After decanting the supernatant, the procedure involved incubating the residue at 60°C for 20 min, along with an additional 3 mL of DMSO. By adding DMSO to the supernatants, the volume was increased to 10 mL. The extracts of chlorophyll and carotenoids were placed in a cuvette (volume: 250 µL) and the absorbance was measured at 663, 645, and 470 nm by an Epoch microplate spectrophotometer (USA) (Ritchie et al., 2021).

#### 2.3.2 Chlorophyll fluorescence

Fluorescence values were measured on fully-expanded leaf samples from 10:00 a.m. until noon. A total number of 7 plants were considered per treatment, using an attachment of pulse-amplitude modulated-leaf fluorometer apparatus (LI-6400, LI-COR, Lincoln, NE, USA). Standard protocols were followed for measuring the fluorescence values (LI-COR 6400 Manual, LI-COR, 2005). After adapting the samples to a dark environment (40 min), assessments were aimed at finding the Fv/Fm value. Fm refers to a maximum value of fluorescence in a dark-adapted state, whereas Fv indicates differences from a maximum to a minimum value of fluorescence in a state of dark adaptation (F0). Fv/Fm refers to the efficiency of PSII at its peak (Bahmanbiglo and Eshghi, 2021).

#### 2.3.3 Gas exchange, photosynthetic rate, and carboxylation efficiency

Several parameters, two months after treatment application, were considered in relation to gas exchange (i.e., transpiration rate, photosynthesis rate, stomatal resistance to CO_2_, and sub-stomatal CO_2_). These factors were measured from 09:00 a.m. to 12:00 using an LCi Portable Photosynthesis System (ADC Bioscientific Ltd). All measurements were carried out at a PPFD value of 1,200 μmol m^−2^ s^−1^, when the temperature of leaf samples was 25°C. The photosynthetic activity of fully-expanded leaf samples was measured on 7 plants in each treatment group. The measurements were carried out from 10:00 a.m. until noon to measure the maximum values. A steady condition of measurable photosynthesis was reached in 3 to 4 min, so that data could be collected. The measurements were also considered on intercellular CO_2_ concentrations (Ci), stomatal conductance to water-vapor, and transpiration rate (E). In practice, the LI-6400 chamber was maintained at an ambient temperature of 26 ± 0.4°C. Airflow occurred at 500 ml min^−1^, while CO_2_ regulation operated at 380 mg/L with the help of a CO_2_ blender. Carboxylation efficiency was expressed as A/Ci protocol (Zhang et al., 2001; Stinziano et al., 2017; Zhou et al., 2019)

#### 2.3.4 Leaf area and anatomical measurements

After scanning the leaf samples by an Epson scanner-device (V550, Epson) with a resolution of 600-dpi, the scanned images assisted in measuring leaf area using MATLAB (version ≥ 2016a) procedure (Huang et al., 2021). At sizes of 5 mm, three leaf disks were dissected from leaf samples and were sampled on a random basis from the plants of each treatment group. A freeze dryer was used for drying the disks before gold sputtering. A 438 VP SEM (Leo Electron Microscopy, Cambridge, UK) was used along with a Centaurus-detector for examining the samples (K.E. Development, Cambridge, UK). Micrographs were randomly taken at various magnifications for each evaluation of trichome and stomatal conditions. The images were analyzed with Image J to measure the length, width, and number of stomatal apertures. In addition to measuring trichome density in the leaf area, the stomatal ultrastructure was measured in fresh leaf samples by scanning electron microscopy (SEM). SEM assessments were performed on fully-grown photosynthetic leaves. The central part of each leaf was sectioned into little segments (1 mm). After fixing the samples in glutaraldehyde (2.5%, 24 h), they were fixed in sodium-sulfide (0.5%, pH 7.2, 30 minutes) to prevent air bubbles from entering. Then, they were rinsed with phosphate buffer (0.1 M, pH 7.2) three times (at 15-minute intervals). This was followed by fixing the samples in Osmium tetroxide (1%) in phosphate buffer (0.1 M, pH 7.2) for 12 h (4°C) before entering the phase of dehydration *via* ethanol. The samples were dehydrated before being imbedded in LR-white and before polymerization (60°C, 24 h). Following the searing of samples under CO_2_, the samples were then gold-sputtered with a JFC-1600 metal-sputtering apparatus and imaged subsequently. The features of stomata in the leaf samples, such as stomatal aperture size and stomata density, were determined in detail while adhering those values of these parameters usually depend on leaf maturity, position, and surface (abaxial or adaxial), as well as stress factors. NIS Element 7.0 software was used for examining the stomatal features (de Oliveira et al., 2022; Khoshravesh et al., 2022).

#### 2.3.5 Electrolyte leakage and membrane stability index

Fresh leaf samples (0.5 g) were taken from young branches of identical size. The samples were washed with sterilized water and then moved to falcon tubes with deionized water (10 mL). Then, the samples were shaken for 24 h at 25°C. This was followed by autoclaving each sample batch at 121°C for 20 min. The electrical conductivity (EC_1_) and ultimate electrical conductivity (EC_2_) were calculated for each solution (Thalhammer et al., 2020). In two test tubes, MSI values were computed by processing the leaf samples (0.1 g) in distilled water (10 ml). One set of test tubes was heated for 30 min inside a water bath (40°C). Accordingly, EC_1_ represented the value of electrical conductivity in the water which contained each sample. After heating the test tubes for a second time, electrical conductivity was measured again (EC_2_) in a water bath (100°C, 15 min). The MSI value was calculated according to a relevant formula (Behdad et al., 2021):


$$\text{Membrane Stability Index}=\left[1-\left(\frac{\text{Electrical conductivity }\left(\text{EC}1\right)\;}{\text{Ultimate electrical conductivity }\left(\text{EC}2\right)}\right)\times 100\right]$$

#### 2.3.6 Malondialdehyde

To quantify the MDA concentration, fresh leaf samples (0.5 g), were placed in trichloroacetic acid (2 mL, 1%) to be homogenized. Then, they were centrifuged for 10 min (10,000 g). The supernatant (250 µL) was poured into trichloroacetic acid (20%, 1 mL) with thiobarbituric acid (0.5%). The solution was submerged in high-temperature water (90°C) for 30 min before decreasing its temperature by an ice bath and before centrifuge. The absorbance values were determined at 600, 532, and 450 nm by a spectrophotometer (Khoubnasabjafari and Jouyban, 2020).

#### 2.3.7 Water use efficiency and relative water content

RWC was calculated using fully-grown leaves per plant sample. Each leaf was weighed (fresh weight-FW) and then saturated with distilled water. The samples were stored at room temperature for 24 h before being shaken. Then, the turgid weight (TW) was obtained by reweighing the samples. Finally, oven-drying the samples took 24 h (60°C), so that the dry weight (DW) could be determined (Badr and Brueggemann, 2020). WUE was expressed as WUE= A/E based on a method by de Santana et al. (2015), A: Photosynthesis rate, E:Transpiration rate (de Santana et al., 2015).


$$\text{Water Use Efficiency}=\frac{\text{Photosynthesis rate }\left(\text{A}\right)}{\text{Transpiration rate }\left(\text{E}\right)}$$

### 2.4 Statistical analysis

Minitab v. 18 software was used for statistical analysis. A completely randomized design was used for conducting the experiments by a factorial pattern with three replicates. The factors were licorice accessions, salinity, and bacterial inoculation. The experimental unit was pot. Analysis of variance (ANOVA) was utilized to analyze the experimental data, and then to apply Tukey’s multiple range test on mean comparisons (*p* ≤ 0.05). The slice approach was employed on mean comparisons where there were significant interactions. Minitab software enabled the execution of principal component analysis (PCA) according to a correlation matrix. Dendrogram was drawn based on Euclidean distance (Minitab; version 17). Corrplot was created using R software version 4.1.1 (Corrplot package).

## 3 Results

### 3.1 Relative water content and water use efficiency

**Figure 2:**
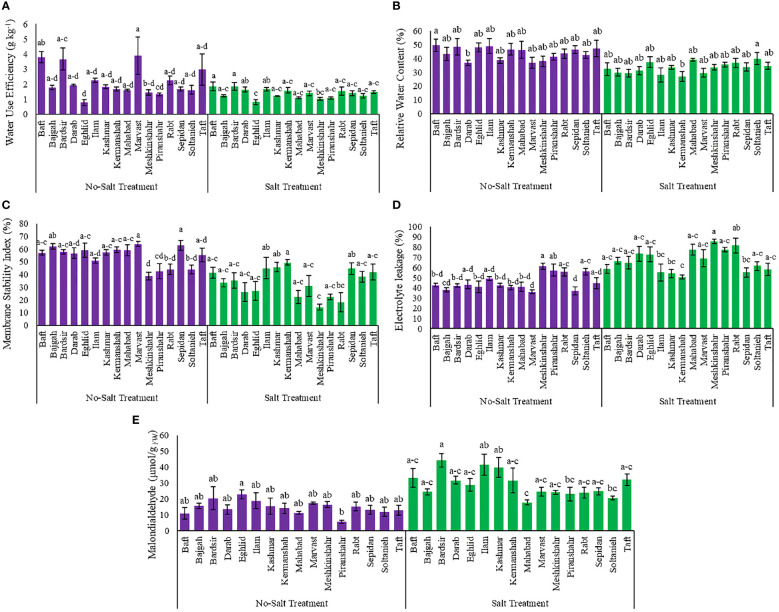
Measured parameters variation under studied treatments. **(A)** Water use efficiency, **(B)** Relative water content, **(C)** Membrane stability index, **(D)** Electrolyte leakage and **(E)** Malondialdehyde. According to the analysis of variance, the above present mean comparisons are the only effects that showed significant difference. Mean values with the same letters are not significantly different (*p* ≤ 0.05), Tukey test. Bars stand for standard error (SE).

Relative water content and water use efficiency affected by different treatments in the current study (**Tables S1**). Salinity reduced the RWC and water use efficiency in plants of different accessions. Maximum reduction in RWC and water use efficiency, due to salinity stress, was observed in Ilam (42.6%) and Marvast (64.2%) accessions, respectively (**Figures 2A, B**). Meanwhile, the minimal decrease in RWC and water use efficiency, because of salinity stress, occurred in Soltanieh (6%) and Kermanshah (5.6%) accessions, respectively (**Figures 2A, B**).

### 3.2 Electrolyte leakage and membrane stability index

The findings showed that the interaction effect between accessions and salinity stress affected significantly electrolyte leakage and membrane stability index (**Table S1**). Data analysis revealed that salinity caused an increase of the electrolyte leakage rate in different accessions but a decrease of the membrane stability index (**Figures 2C, D**). The lowest value of electrolyte leakage was observed in the Kermanshah accession (50.68%), while its cell membranes had the strongest stability against salinity stress (49.3%) (**Figures 2C, D**). The Soltanieh accession had a minimal increase (10%) in electrolyte leakage, compared to the other accessions (**Figure 2D**).

### 3.3 Malondialdehyde

The analysis of variance showed that integrated salinity stress and accessions affected MDA content significantly (**Table S1** and **Figure 2E**). As the salinity negatively affected membrane lipid peroxidation, minimum enhancement in MDA content under salinity stress was observed in the Eghlid accession (26.6%) while maximum increase was achieved in Piranshahr accession (4 folds) (**Figure 2E**).

### 3.4 Photosynthetic pigments

**Figure 3:**
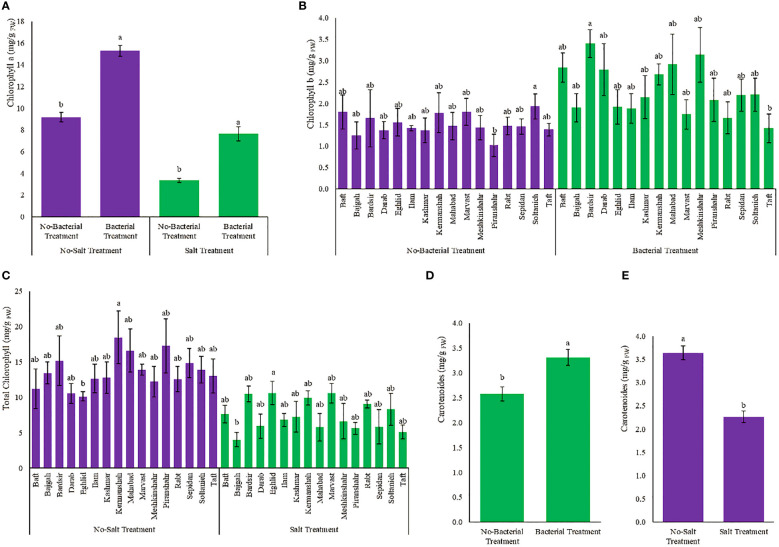
Measured parameters variation under studied treatments. **(A)** Chlorophyll a, **(B)** Chlorophyll b, **(C)** Total chlorophyll and **(D, E)** Carotenoid. According to the analysis of variance, the above present mean comparisons are the only effects that showed significant difference. Mean values with the same letters are not significantly different (*p* ≤ 0.05), Tukey test. Bars stand for standard error (SE).

Photosynthetic pigments diversely affected by various treatments (**Table S2**). The presence of bacteria, despite salinity, increased chlorophyll a content which became 2.3 folds higher than the condition of having salinity without bacteria (**Figures 3A, B**). Meshkinshahr (2.2 folds) and Bardsir (2 folds) accessions showed maximum increase in chlorophyll b under bacterial inoculation (**Figures 3A, B**). Minimum total chlorophyll content was observed in Bajgah accession, under salinity stress, which was 70% less than the amount in the corresponding control without salinity (**Figure 3C**). Minimum reduction in total chlorophyll under salinity stress achieved in Marvast (23.9%) and Rabt (27.8%) accessions (**Figure 3C**). The maximum amount of carotenoids occurred as a result of bacterial inoculation and became 28.3% higher than the non-bacterial treatment (**Figure 3D**). Salinity stress reduced carotenoids (37.7%) (**Figure 3E**). Chlorophyl a/b ratio, and Total chlorophyll/Carotenoids ratio are also presented in **Figures S1**, **S2**, **S3**, **S4**.

### 3.5 Chlorophyll fluorescence

**Figure 4:**
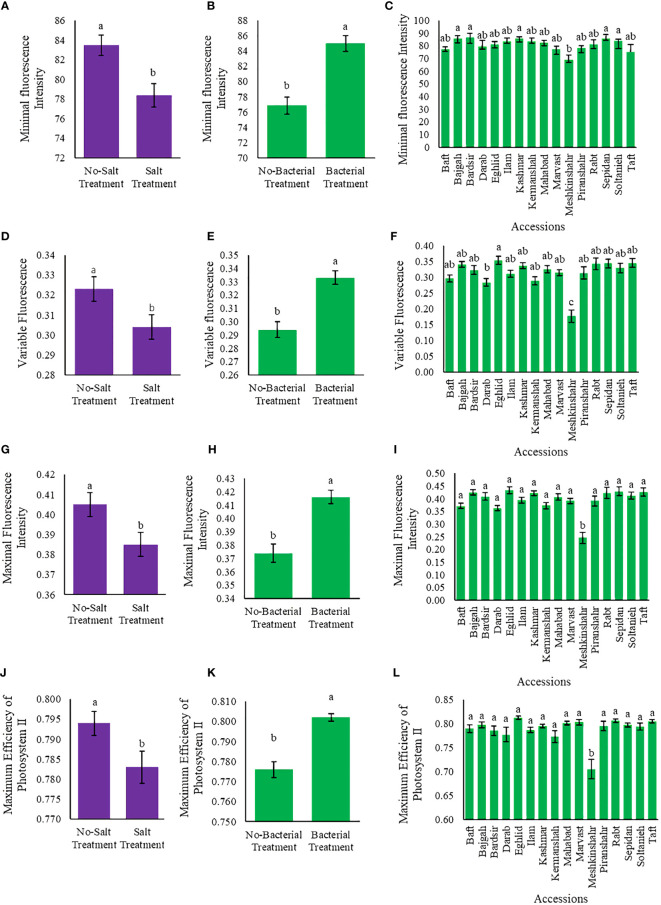
Measured parameters variation under studied treatments. Variable fluorescence (Fv), Maximal fluorescence intensity (Fm), and Maximum quantum yield **(A–C)** Minimal fluorescence intensity (F0), **(D–F)** Variable fluorescence (Fv), **(G–I)** Maximal fluorescence intensity (Fm) **(J–L)** Maximum quantum yield (Fv/Fm). According to the analysis of variance, the above present mean comparisons are the only effects that showed significant difference. Mean values with the same letters are not significantly different (*p* ≤ 0.05), Tukey test. Bars stand for standard error (SE).

Chlorophyll fluorescence parameters varied among applied treatments (**Tables S2**, **S3**). Bacteria and salinity affected the values of chlorophyll fluorescence parameters (F0, Fv, Fm, and Fv/Fm) in opposite ways so that when the amount of bacteria increased, salinity stress decreased. Maximum F0 (86.7) was observed in Bardsir accession, while the highest Fv, Fm, and Fv/Fm values (i.e., 0.35, 0.43, and 0.81, respectively) were observed in Eghlid accession (**Figures 4A–L**). The least Fv/Fm value was observed in Meshkinshahr accession (0.705) (**Figure 4L**). Salinity stress decreased Fv value (5.9%) while bacterial inoculation increased Fv (13.3%) (**Figures 4D, E**). Also, Fv/Fm, Fm/Fo, and Fv/Fo ratios are presented in **Figures S5**–**S9**.

### 3.6 Gas exchange and photosynthetic rate

Gas exchange and photosynthetic rate diversely affected by bacterial inoculation and salinity stress in different accessions (**Tables S3**). Bacterial inoculation mitigated salinity stress while preventing the decrease in stomatal conductance and substomatal CO_2_ in various accessions. The concurrence of salinity stress and bacterial inoculation caused an increase in the values of these parameters in the different accessions. The highest increase in stomatal CO_2_ was recorded in Baft and Ilam accessions (25.8% and 25.6%, respectively) (**Table 3**), and the highest increase in stomatal conductance was recorded in Bardsir accession (3 folds) (**Table 3**). Salinity stress and bacterial inoculation affected photosynthetic rates in all accessions. The results showed that salinity stress reduced the rate of photosynthesis. Among the accessions, Kermanshah and Sepidan demonstrated the sharpest decrease in photosynthetic rate (60.5% and 28.6%, respectively) (**Table 3**). However, under bacterial inoculation, the negative effects of salinity stress on photosynthetic rate were mitigated. Plants treated with bacterial inoculation and salinity stress showed higher photosynthetic rates than those treated with salinity stress only. The highest values of photosynthetic rate were recorded in Meshkinshahr and Kermanshah accession, which were 2.4 folds and 2 folds higher, respectively, compared to the salt-stressed control group without bacterial inoculation (**Table 3**).

**Table 3:** Effect of *Azotobacter* and salinity stress interactions on measured photosynthetic parameters of 16 Iranian licorice accessions.

Traits													
		Photosynthetic Rate(µmol m^-2^ s^-1^)				Stomatal Conductance of CO_2_(Mol m^-2^ s^-1^)			Sub-Stomatal CO_2_(vpm)				
		No-Bacterial treatment		Bacterial treatment		No-Bacterial treatment	Bacterial treatment		No-Bacterial treatment		Bacterial treatment		
No.	Accessions	No-Salt treatment	Salt treatment	No-Salt treatment	Salt treatment	No-Salt treatment	Salt treatment	No-Salt treatment	Salt treatment	No-Salt treatment	Salt treatment	No-Salt treatment	Salt treatment
1	Baft	2.86^ab^ ± 0.2	2.98^a-c^ ± 0.55	8.8^ab^ ± 1.06	2.54^a-b^ ± 0.1	0.06^c^ ± 0.03	0.035^bc^ ± 0.008	0.135^ab^ ± 0.095	0.085^ab^ ± 0.045	261.3^b^ ± 15	192.3^c^ ± 6	270^de^ ± 2.8	242^bc^ ± 8.6
2	Bajgah	5.42^ab^ ± 0.69	2.56^a-c^ ± 0.13	5.92^b-d^ ± 0.11	4.07^a-b^ ± 1.43	0.08^c^ ± 0.031	0.07^bc^ ± 0.005	0.095^b^ ± 0.02	0.085^ab^ ± 0.002	255.3^b^ ± 4	220^a-c^ ± 1	275.3^c-e^ ± 7.8	235.3^c^ ± 8.3
3	Bardsir	7.05^a^ ± 1.71	2.81^a-c^ ± 0.31	10.67^a^ ± 1.14	3.89^a-b^ ± 1.65	0.08^c^ ± 0.017	0.025^c^ ± 0.008	0.123^ab^ ± 0.065	0.076^ab^ ± 0.041	300.3^ab^ ± 17	240^a-c^ ± 25	300.6^b-e^ ± 7.5	267.6^a-c^ ± 3.1
4	Darab	4.62^ab^ ± 1	2.82^a-c^ ± 0.19	7.12^a-d^ ± 0.23	4.24^a-b^ ± 0.45	0.09^c^ ± 0.014	0.07^bc^ ± 0	0.12^ab^ ± 0.011	0.075^ab^ ± 0.014	256.3^b^ ± 18	248.3^a-c^ ± 2.6	264.3^e^ ± 7.2	249^bc^ ± 6.9
5	Eghlid	2.84^b^ ± 0.77	1.4^c^ ± 0.14	3.63^d^ ± 0.79	2.69^a-b^ ± 0.83	0.16^c^ ± 0.017	0.052^bc^ ± 0.021	0.47^a^ ± 0.225	0.055^ab^ ± 0.008	317^a^ ± 12	287.3^a^ ± 9.5	321.3^a-c^ ± 10.1	309.3^a^ ± 6
6	Ilam	5.04^ab^ ± 0.37	2.14^c^ ± 0.14	5.69^b-d^ ± 0.33	4.27^a-b^ ± 0.41	0.11^c^ ± 0.017	0.025^c^ ± 0.002	0.125^ab^ ± 0.014	0.055^ab^ ± 0.008	262.3^b^ ± 2	195.3^c^ ± 3	264^e^ ± 15.6	245.3^bc^ ± 13
7	Kashmar	6.07^ab^ ± 0.16	3.18^a-c^ ± 0.64	6.32^b-d^ ± 0.86	4.49^a-b^ ± 0.01	0.13^c^ ± 0.034	0.065^bc^ ± 0.002	0.18^ab^ ± 0.023	0.065^ab^ ± 0.014	263.6^b^ ± 4	222.3^a-c^ ± 7.8	300.3^b-e^ ± 3.4	242^bc^ ± 6.9
8	Kermanshah	5.55^ab^ ± 0.92	2.19^bc^ ± 0.34	7.92^a-c^ ± 1.13	4.7^a-b^ ± 0.23	0.09^c^ ± 0.023	0.055 ^bc^ ± 0.008	0.28^ab^ ± 0.092	0.09^ab^ ± 0.005	275.3^ab^ ± 10	229^a-c^ ± 12.3	285^b-e^ ± 1.1	261^a-c^ ± 9.2
9	Mahabad	6.76^ab^ ± 0.14	4.4^a^ ± 0.19	6.71^b-d^ ± 0.71	5.35^a-b^ ± 0	0.15^c^ ± 0.011	0.085^a-c^ ± 0.002	0.245^ab^ ± 0.026	0.125^ab^ ± 0.008	299^ab^ ± 8	234^a-c^ ± 1.7	295^b-e^ ± 0.57	263.3^a-c^ ± 6.6
10	Marvast	4.19^ab^ ± 0.71	1.85^c^ ± 0.28	5.12^c-d^ ± 0.41	1.9^b^ ± 0.04	0.04^c^ ± 0.02	0.03^bc^ ± 0.005	0.078^b^ ± 0.016	0.04^b^ ± 0	272^ab^ ± 4	257^a-c^ ± 23.7	318.3^a-d^ ± 11.2	261.3^a-c^ ± 1.4
11	Meshkinshahr	4.13^ab^ ± 0.23	1.67^c^ ± 0.23	7.99^a-c^ ± 0.45	3.9^a-b^ ± 0.47	0.15^c^ ± 0.011	0.11^ab^ ± 0.011	0.18^ab^ ± 0.04	0.145^ab^ ± 0.043	300.3^ab^ ± 5	260.6^a-c^ ± 9.9	326.3^ab^ ± 8.4	294^ab^ ± 10.4
12	Piranshahr	6.17^ab^ ± 0.24	3.1^a-c^ ± 0.15	9^ab^ ± 0.27	5.7^a^ ± 1.64	0.38^b^ ± 0.028	0.11^ab^ ± 0.017	0.405^ab^ ± 0.077	0.156^ab^ ± 0.052	302^ab^ ± 2	270^ab^ ± 15.6	323.6^a-c^ ± 3.7	286.6^a-c^ ± 7.5
13	Rabt	4.76^ab^ ± 0.55	4.14^ab^ ± 0.94	5.58^b-d^ ± 0.37	4.5^a-b^ ± 0.79	0.11^c^ ± 0.028	0.08^a-c^ ± 0.04	0.275^ab^ ± 0.066	0.1^ab^ ± 0.011	284.3^ab^ ± 6	260^a-c^ ± 20.8	358.3^a^ ± 7.2	268^a-c^ ± 8
14	Sepidan	5.72^ab^ ± 0.36	2.27^bc^ ± 0.36	7.56^a-c^ ± 0.22	3.97^a-b^ ± 0.6	0.11^c^ ± 0.026	0.035^bc^ ± 0.002	0.17^ab^ ± 0.017	0.08^ab^ ± 0.017	274.3^ab^ ± 0.8	247^a-c^ ± 11	290.3^b-e^ ± 24	261^a-c^ ± 4.6
15	Soltanieh	7.52^a^ ± 1.19	4.42^a^ ± 0.15	7.24^a-c^ ± 0.86	5.58 ^a-b^ ± 0.23	0.73^a^ ± 0.065	0.16^a^ ± 0.017	0.26^ab^ ± 0.005	0.18^a^ ± 0.063	287^ab^ ± 1.7	262^a-c^ ± 26	290.3^b-e^ ± 4.9	271^a-c^ ± 27.7
16	Taft	4.93^ab^ ± 0.76	3.34^a-c^ ± 0.17	5.14^cd^ ± 0.45	4.05^a-b^ ± 0.45	0.07^c^ ± 0.005	0.055^bc^ ± 0.026	0.105^ab^ ± 0.008	0.06 ^ab^ ± 0.008	256.3^b^ ± 2.6	209^bc^ ± 6.6	266.3^e^ ± 2.6	239.3^c^ ± 3.1

According to the analysis of variance, the triple effects of Azotobacter, salinity, and accessions showed a significant difference, the slice method was used for mean comparisons. Mean values with the same letters within a column are not significantly different (p ≤ 0.05), Tukey test. Means ± standard error (SE).

### 3.7 Leaf area, transpiration rate, and carboxylation efficiency

**Figure 5:**
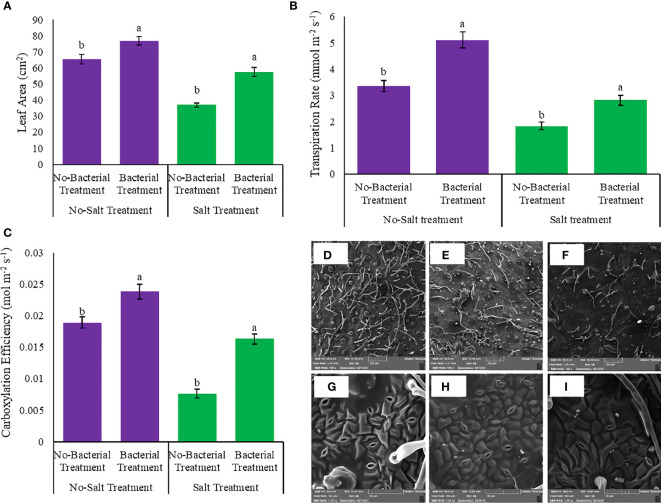
Measured parameters variation under studied treatments. **(A)** Leaf area, **(B)** Transpiration rate, **(C)** Carboxylation efficiency, **(D)** Trichome density under salinity stress **(E)** Trichome density under integrated salinity and bacterial inoculation, **(F)** Trichome density under bacterial inoculation. **(G)** Stomata density under bacterial inoculation, **(H)** Stomata density under salinity stress integrated bacterial inoculation and **(I)** Stomata density under salinity stress. According to the analysis of variance, the above present mean comparisons are the only effects that showed significant difference. Mean values with the same letters are not significantly different (*p* ≤ 0.05), Tukey test. Bars stand for standard error (SE).

Leaf area, transpiration rate, and carboxylation efficiency had showed various responses under studied treatments (**Tables S3**, **S4**). The interaction between salinity and *Azotobacter* had a significant effect on leaf area, transpiration rate, and carboxylation efficiency. Bacterial inoculation caused maximum leaf area, transpiration rate, and carboxylation efficiency, whereas salinity had a negative effect on these parameters (**Figure 5**). Bacterial inoculation increased leaf area under both no salinity (17.3%) and salinity treatment (55.7%) (**Figure 5A**). Transpiration rate also increased by bacterial inoculation, 52.08 and 53.3%, under no salinity and with salinity stress, respectively (**Figure 5B**). Bacterial treatment integrated salinity enhanced carboxylation efficiency (2 folds) towards no bacterial inoculation (**Figure 5C**).

### 3.8 Leaf anatomical measurements

Leaf anatomical characteristics affected under studied treatments (**Table S4**). Salinity reduced the density of stomata and increased the density of trichomes (**Table 4**). Under bacterial inoculation, however, the effects of salinity stress were mitigated. In the present study, the combination of bacterial inoculation and salinity increased stomatal density, but reduced trichome density, compared with no bacterial inoculation. According to the results, the highest increase in stomatal density was observed in the Mahabad accession (2.3 folds), while the maximum decrease in trichome density occurred in the Mahabad accession (70.5%) (**Table 5** and **Figure 5**). Through the effect of bacterial inoculation under salinity stress, stomatal length and width increased compared to no bacterial inoculation. Maximum increases in stomatal length and width were observed in Bajgah and Bardsir accessions, respectively (**Table 5**).

**Table 4:** Effect of *Azotobacter* and salinity stress interactions on number of stomatal and trichome of 16 Iranian licorice accessions.

Traits									
		Number of Stomatal (number/cm^2^)					Number of Trichome (number/cm^2^)			
		No-Bacterial treatment			Bacterial treatment		No-Bacterial treatment		Bacterial treatment	
	No.	Accessions	No-Salt treatment	Salt treatment	No-Salt treatment	Salt treatment	No-Salt treatment	Salt treatment	No-Salt treatment	Salt treatment
	1	Baft	312^gh^ ± 22	377^a^ ± 22	611^de^ ± 34	273^d^ ± 22	40^c-e^ ± 5.77	120^cd^ ± 5.77	40^cd^ ± 2.89	75^c^ ± 5.77
	2	Bajgah	455^ef^ ± 13	377^a^ ± 13	1040^ab^ ± 34	390^bc^ ± 22	38^c-f^ ± 1.67	90^ef^ ± 2.89	13^e^ ± 1.67	60^c-e^ ± 2.89
	3	Bardsir	377^fg^ ± 34	351^a^ ± 13	416^g^ ± 13	156^e^ ± 22	32^d-g^ ± 7.26	47^hi^ ± 7.26	25^de^ ± 5.77	43^e-g^ ± 1.67
	4	Darab	351^f-h^ ± 22	351^a^ ± 13	455^fg^ ± 13	299^cd^ ± 13	28^d-g^ ± 1.67	70^f-h^ ± 2.89	18^e^ ± 1.67	65^cd^ ± 2.89
	5	Eghlid	624^cd^ ± 22	351^a^ ± 22	1040^ab^ ± 13	559^a^ ± 13	75^b^ ± 2.89	175^b^ ± 2.89	75^a^ ± 2.89	115^b^ ± 2.89
	6	Ilam	273^gh^ ± 22	351^a^ ± 22	364^g^ ± 13	260^d^ ± 13	20^e-g^ ± 2.89	50^g-i^ ± 2.89	20^e^ ± 2.89	25^gh^ ± 2.89
	7	Kashmar	793b ± 34	299^ab^ ± 22	1001^a-c^ ± 13	624^a^ ± 22	178^a^ ± 4.41	208^a^ ± 10.1	63^ab^ ± 1.67	147^a^ ± 7.26
	8	Kermanshah	702^bc^ ± 22	234^bc^ ± 22	1053^ab^ ± 22	221^de^ ± 13	40^c-e^ ± 2.89	145^c^ ± 2.89	20^e^ ± 2.89	60^c-e^ ± 2.89
	9	Mahabad	533^de^ ± 13	234^bc^ ± 34	546^ef^ ± 22	416^b^ ± 13	18fg ± 1.67	95^d-f^ ± 5.77	18^e^ ± 1.67	28^gh^ ± 1.67
	10	Marvast	546^de^ ± 45	221^bc^ ± 13	676^d^ ± 13	403^b^ ± 34	72^b^ ± 4.41	120^cd^ ± 7.64	63^ab^ ± 7.26	77^c^ ± 4.41
	11	Meshkinshahr	221^h^ ± 13	221^bc^ ± 13	676^d^ ± 13	143^e^ ± 13	15^g^ ± 2.89	25^i^ ± 2.89	13^e^ ± 1.67	24^h^ ± 1.67
	12	Piranshahr	650^cd^ ± 13	182^c^ ± 22	897^c^ ± 22	429^b^ ± 22	55^bc^ ± 2.89	80^ef^ ± 2.89	30^de^ ± 2.89	58^c-f^ ± 4.41
	13	Rabt	312^gh^ ± 22	156^c^ ± 22	351^g^ ± 22	299^cd^ ± 13	25^d-g^ ± 2.89	70^f-h^ ± 2.89	20e ± 2.89	29^gh^ ± 4.41
	14	Sepidan	546^de^ ± 22	156^c^ ± 22	624^de^ ± 22	533^a^ ± 13	25^d-g^ ± 2.89	75^fg^ ± 2.89	13^e^ ± 1.67	40^f-h^ ± 2.89
	15	Soltanieh	1040^a^ ± 34	143^c^ ± 13	1092^a^ ± 45	624^a^ ± 22	45^cd^ ± 2.89	70^f-h^ ± 2.89	18^e^ ± 1.67	52^d-f^ ± 4.41
	16	Taft	312^gh^ ± 22	130^c^ ± 13	949^bc^ ± 13	273^d^ ± 22	55^bc^ ± 5.77	103^de^ ± 4.41	50^bc^ ± 2.89	65^cd^ ± 2.89

According to the analysis of variance, the triple effects of Azotobacter, salinity, and accessions showed a significant difference, the slice method was used for mean comparisons. Mean values with the same letters within a column are not significantly different (p ≤ 0.05), Tukey test. Means ± standard error (SE).

**Table 5:** Effect of *Azotobacter* and salinity stress interactions on measured stomatal characteristics of 16 Iranian licorice accessions.

Traits										
		Stomatal Length(µm)				Stomatal Width(µm)			
		No-Bacterial treatment		Bacterial treatment		No-Bacterial treatment		Bacterial treatment		
No.	Accessions	No-Salt treatment	Salt treatment	No-Salt treatment	Salt treatment	No-Salt treatment	Salt treatment	No-Salt treatment	Salt treatment
1	Baft	13.05^f-h ±^0.86	8.99^gh ±^0.26	14.49 ± 1.23	11.61^f ±^0.22	2.29^bc ±^0.31	1.3^fg ±^0.01	3.4^c-e ±^0.09	1.97^c-e ±^0.15
2	Bajgah	13.58^e-h ±^0.7	8.35^h ±^0.32	14.62 ± 0.21	13.49^b-d ±^0.11	3.34^ab ±^0.1	3.13^a ±^0.41	3.44^c-e ±^0.2	3.3^ab ±^0.2
3	Bardsir	13.39^e-h ±^0.1	10.46^e-g ±^0.13	16.16 ± 0.09	13.22^c-e ±^0.46	3.27^ab ±^0.32	1.04^g ±^0.02	3.63^b-d ±^0.08	2.75^a-c ±^0.05
4	Darab	16.17^a-d ±^0.21	10.36^fg ±^0.6	16.74 ± 0.37	14.52^b ±^0.12	2.5^a-c ±^0.1	2.26^b-d ±^0.04	2.94^de ±^0.03	2.43^b-d ±^0.16
5	Eghlid	14.29^d-g ±^0.34	12.67^b-d ±^0.17	14.9 ± 0.25	14.14^bc ±^0.16	3.07^ab ±^0.37	1.37^fg ±^0.22	4.94^a ±^0.42	2.79^a-c ±^0.28
6	Ilam	11.46^h ±^0.9	9.85^fg ±^0.17	14.63 ± 0.14	10.1^g^ ± 0.38	2.62^a-c ±^0.19	2.45^a-d ±^0.01	3.11^c-e ±^0.21	2.52^a-d ±^0.12
7	Kashmar	15.28^c-f ±^0.12	12.15^c-e ±^0.19	15.61 ± 0.189	14.02^bc ±^0.18	3.25^ab ±^0.16	2.63^a-c ±^0.14	3.3^c-e ±^0.15	3.21^ab ±^0.09
8	Kermanshah	14.49^d-g ±^0.07	13.12^b-d ±^0.6	14.49 ± 0.53	13.23^c-e ±^0.08	3.37^ab ±^0.2	2.39^a-d ±^0.11	3.95^bc ±^0.04	2.43^b-d ±^0.3
9	Mahabad	13.95 ± 0.03	11.44^d-f ±^0.21	15.83 ± 0.17	12.63^d-f ±^0.31	2.56^a-c ±^0.17	1.87^c-f ±^0.11	2.86^de ±^0.08	2.08^c-e ±^0.04
10	Marvast	13.62^e-h ±^0.23	8.77^gh ±^0.39	13.6167 ± 0.2	12.56^ef ±^0.18	3.54^a ±^0.12	1.52^e-g ±^0.18	4.36^ab ±^0.12	2.58^a-d ±^0.09
11	Meshkinshahr	17.85^a ±^0.39	16.21^a ±^0.03	19.33 ± 0.73	16.8^a ±^0.12	2.44^a-c ±^0.11	0.97^g ±^0.03	2.85^de ±^0.08	1.72^de ±^0.25
12	Piranshahr	16.13^a-d ±^0.01	13.49^bc ±^0.17	17.33 ± 0.19	13.87^b-d ±^0.06	3.11^ab ±^0.04	2.95^ab ±^0.03	3.51^b-e ±^0.22	3.03^ab ±^0.04
13	Rabt	12.61^gh ±^0.36	8.16^h ±^0.46	13.61 ± 0.08	11.89^f ±^0.38	1.93^c ±^0.38	1.52^e-g ±^0.15	2.66^e ±^0.17	1.56^e ±^0.07
14	Sepidan	15.54^b-e ±^0.23	14.32^b ±^0.26	15.75 ± 0.37	14.37^bc ±^0.17	3.16^ab ±^0.08	2.5^a-d ±^0.08	3.3^c-e ±^2.11	2.54^a-d ±^0.23
15	Soltanieh	16.75^a-c ±^0.4	13.34^bc ±^0.07	19.46 ± 0.11	15.82^a ±^0.24	3.43^a ±^0.13	2.57^a-c ±^0.17	3.54^b-e ±^0.18	3.4^a ±^0.11
16	Taft	17.61^ab ±^0.24	12.84^b-d ±^0.53	17.83 ± 0.37	13.67^b-d ±^0.19	3.45^a ±^0.13	1.74^d-g ±^0.14	3.36^c-e ±^0.12	2.62^a-d ±^0.15

According to the analysis of variance, the triple effects of Azotobacter, salinity, and accessions showed a significant difference, the slice method was used for mean comparisons. Mean values with the same letters within a column are not significantly different (p ≤ 0.05), Tukey test. Means ± standard error (SE).

### 3.9 Principal component, correlation, and cluster analysis of measured parameters of Glycyrrhiza glabra accessions under integrated Azotobacter inoculation and salinity stress

**Figure 6:**
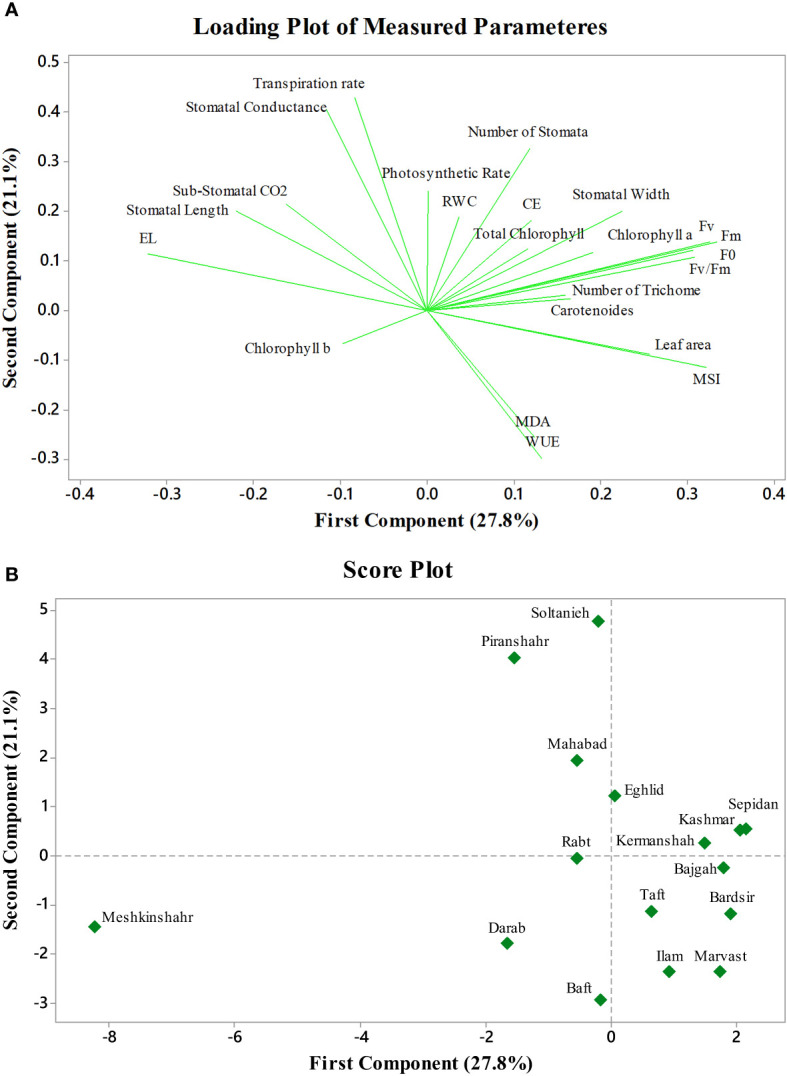
Principal component analysis of Azotobacter and salinity stress on 16 licorice accessions. **(A)**: Loading plot of measured parameters. **(B)**: Score plot of studied accessions. CE (Carboxylation Efficiency), EL (Electrolyte Leakage), MSI (Membrane Stability Index), MDA (Malondialdehyde), WUE (Water Use Efficiency), RWC (Relative water content), F0 (Initial fluorescence intensity), Fv (Variable fluorescence), and Fm (Maximal fluorescence intensity).

**Figure 7:**
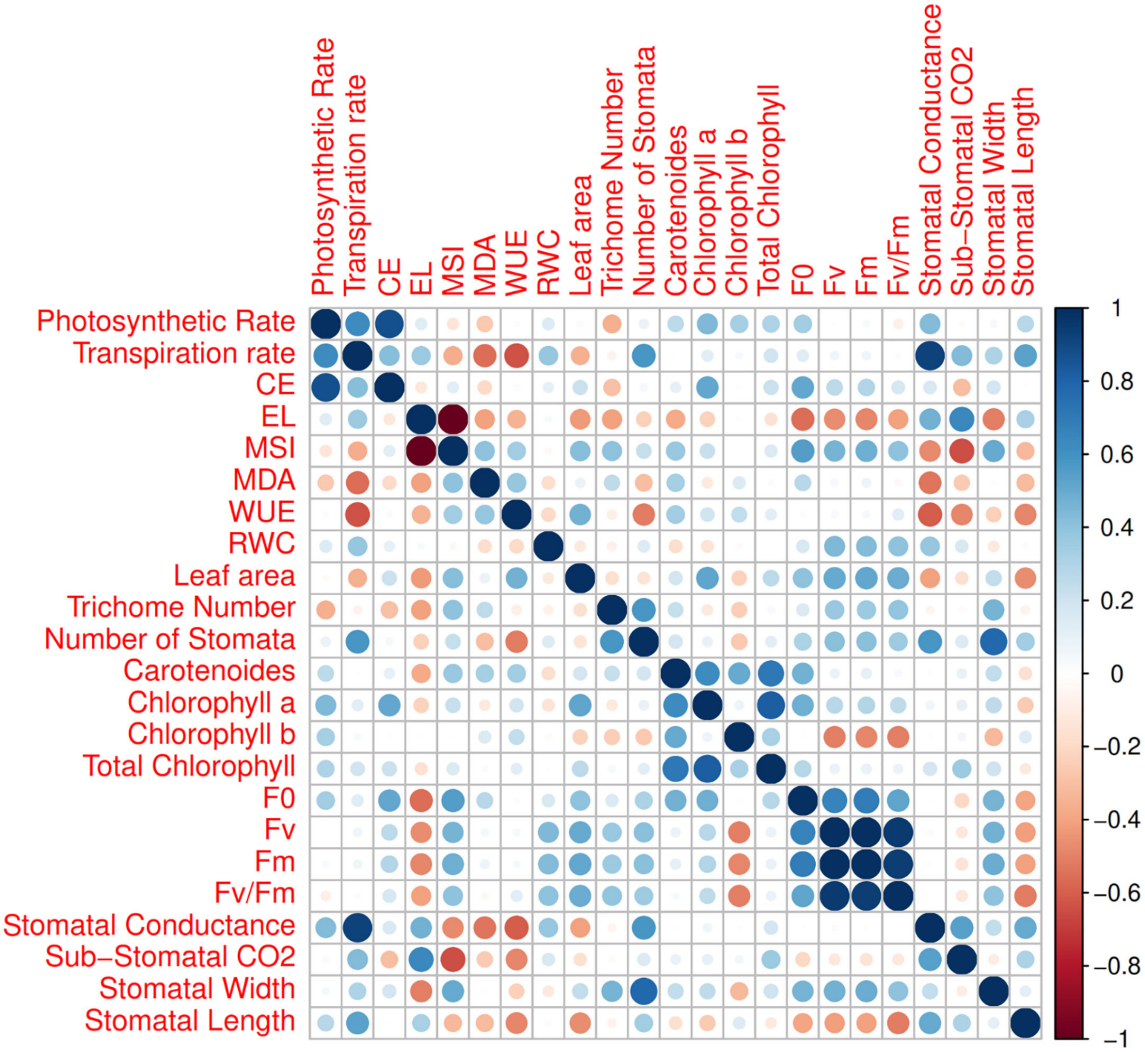
Corrplot between various measured parameters of 16 Iranian licorice accessions. CE (Carboxylation Efficiency), EL (Electrolyte Leakage), MSI (Membrane Stability Index), MDA (Malondialdehyde), WUE (Water Use Efficiency), RWC (Relative water content), F0 (Initial fluorescence intensity), Fv (Variable fluorescence), and Fm (Maximal fluorescence intensity).

The first two PCs in the biplot of PCA explained 48.9% of the differences in characteristics between the treatment groups (**Figure 6**). The first PC, which included photosynthetic pigments, chlorophyll fluorescence parameters, MSI, leaf area, MDA, and the number of stomata explained 27.8% of the variation. The second PC, on the other hand, accounted for 21.1% of the differences in features, comprising stomatal conductance, transpiration rate, and EL. The biplot projection of treatments on the two PCs in this research revealed that the genotypes were separated into several groups. The first group comprised ten genotypes, i.e., Marvast, Ilam, Bardsir, Taft, Kermanshah, Bajgah, Sepidan, Kashmar, as categorized concerning the first PC-related features. The results showed that each treatment group that was dispersed over the PC vectors performed better (**Figure 6**). Using correlation analysis, successful evaluations showed positive correlations among licorice photosynthetic traits, including photosynthetic rate, carboxylation efficiency, transpiration rate, and stomatal conductance (**Figure 7**). Among the measured parameters, MSI showed a negative correlation with EL (35.2%) (**Figure 7**).

**Figure 8:**
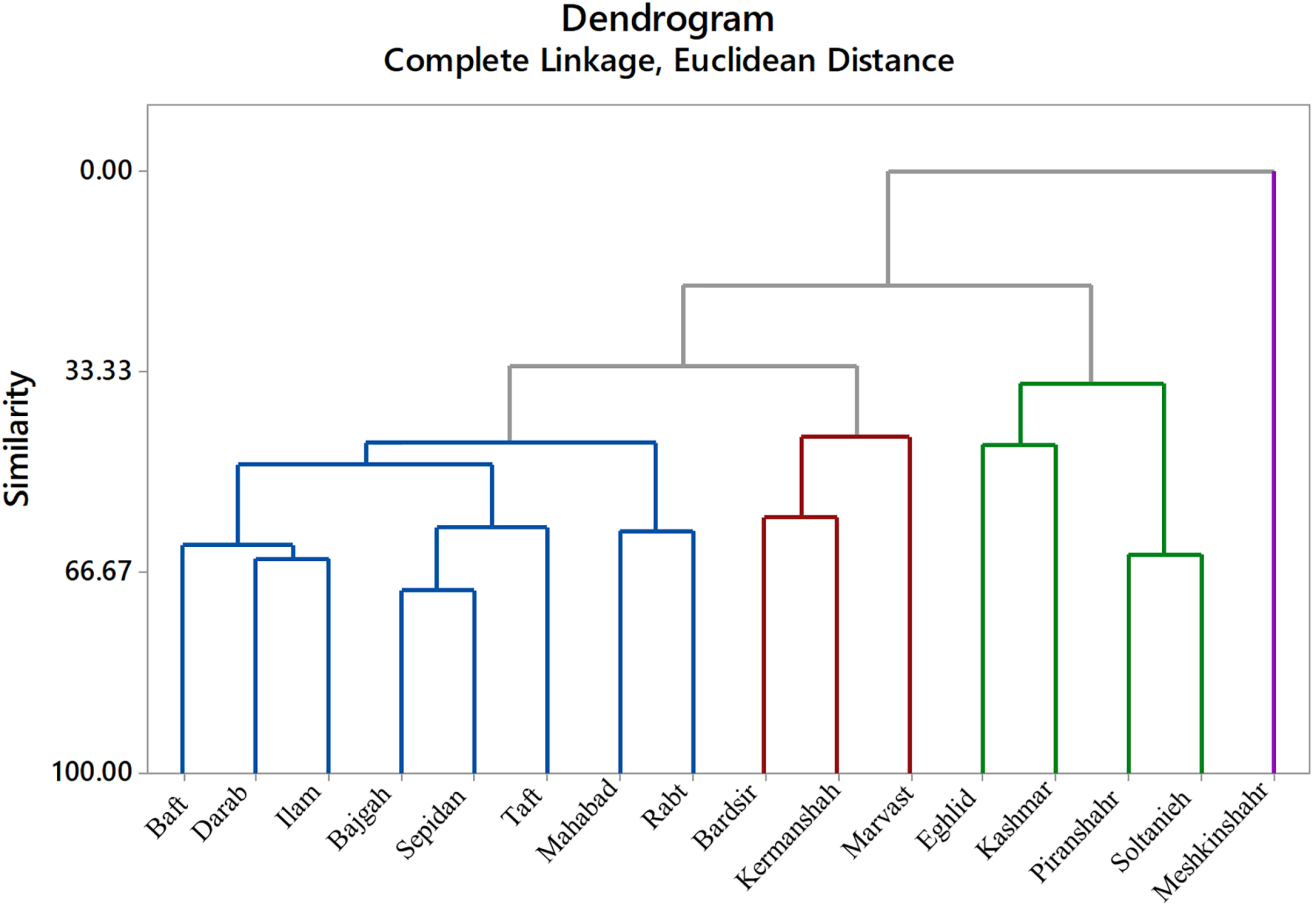
Cluster analysis of Iranian licorice accessions under studied treatments.

The cluster analysis was done based on complete linkage and Euclidean distance into four separated groups (**Figure 8**). Meshkinshahr accession separated into a distinct group regarding photosynthetic measurements. Bardsir, Kermanshah, and Marvast were also categorized in a separate cluster.

## 4 Discussion

Plant growth depends on photosynthesis and is usually susceptible to abiotic stress. To relieve stress, plants manage the exit of water vapor by modifying the stomatal opening and through the growth/expansion of leaf area. When plants are first exposed to salt stress, they limit the amount of water loss from leaves primarily by slowing the rate of transpiration by stomatal closure, although this reduces the capability of CO_2_ uptake and is followed by osmotic stress (Correia et al., 2021; Mousavi et al., 2022). Although stomatal closure is one of the most common causes of the decrease in photosynthetic rates, metabolic constraints such as lowered levels of Rubisco activity/content can also induce a decrease in photosynthetic rates (Zunzunegui et al., 2021; Singh et al., 2022). Higher osmotic tolerance causes leaf expansion and stomatal conductance (Better stomatal conductance leads to optimal transfer of water and nutrients. These substances increase plant growth and leaf development. Also, enough water causes cell enlargement, which occurs after improving the osmotic conditions.), which is beneficial only when there is enough moisture in the soil to compensate for the loss of moisture through transpiration (Ilangumaran and Smith, 2017; Zunzunegui et al., 2021). Salinity affected photosynthesis also by nonstomatal factors that involved the inhibition of the electron transport chain, damage of oxygen evolving complex, reduction of electron transferring capability at donor-sides of PSII, and inactivation of PSII reaction centers (Kalaji et al., 2018). Similar to the current results, previous research on rice and wheat showed that salinity caused a drop in osmotic-potentials of leaves, photosynthetic rates, electron transport rates, and CO_2_ levels in chloroplasts, which were associated with significant decreases in the quantum yield of PSII and total chlorophyll content (Hussain et al., 2021). After exposure to salinity, it is usual that the photosynthetic rate decreases in response to a decline in leaf area, gas exchange, and also feedback suppression of unused photosynthates (Ilangumaran and Smith, 2017). As observed in the present study, under the effect of salinity, PGPB led to a higher photosynthetic efficiency in licorice plants (by increasing osmolyte production, reduction in membrane damage, higher antioxidant activity, all to protect photosynthetic apparatus) (Rossi et al., 2021), as were similar cases observed in maize plants (*Zea mays* L.) (Hafez et al., 2021) and pistachio trees (*Pistacia vera*) (Khalilpour et al., 2021).

Chlorophyll, a primary chloroplast component, absorbs active radiation in plants through photosynthesis, while accessory pigments assist in this regard by chlorophyll-protein complexes. One mechanism by which chlorophyll molecules are protected against membrane damage is through the activity of carotenoids which operate by harvesting light, as well as preventing oxygen molecules from the possibility of molecular excitation in oxygen-chlorophyll complexes, associated with quenching triplet-chlorophyll molecules (Junior et al., 2021). Reactive oxygen species (ROS) tend to degrade chlorophyll pigments rapidly and make them oxidized, thereby impairing the biosynthesis of pigments, followed by a lowered rate of biosynthesis in chlorophyll molecules, as well as enhanced levels of chlorophyllase activity in breaking down chlorophyll, and enabling interactions between Cl^-^ and Na^+^ ions while affecting protein-pigment complexes. All of these factors can contribute to a decline in photosynthetic pigments within the rhizosphere (Keshavarz and Moghadam, 2017; Sarabi et al., 2017). In agreement with the present results, research on strawberry (*Fragaria* × *ananassa* Duchesne) showed that salinity stress affected the ratios of pigments from photosynthetic activity (Avestan et al., 2021). This was similarly observed in the case of Amaranth (*Amaranthus tricolor* L.) (Siswanti and Umah, 2021). Salinity stress tends to reduce photosynthetic rates in plants by causing high levels of accumulated Na^+^ and/or Cl^-^, as well as a lowered level of water potential in plants, all of which have a direct effect on plant health (Afridi et al., 2019). A decrease in pigment content can be explained by mechanisms that assist in reducing photoinhibition and in avoiding the stress factors that inhibit pigment synthesis (Choinski et al., 2003). Several studies have found that halotolerant PGPB improves the growth of a variety of crops under salinity stress. PGPB is assumed as a stimulant of growth *via* activating antioxidant defense in plants, contributing to the fixation of atmospheric nitrogen, helping to solubilize K and P, and generating siderophores. Also, it reduces the accumulation of toxic ions and improves the nutritional status of nutrients. PGPB can lower Na^+^ buildup in plants by releasing EPS to become bound with cations (particularly Na^+^) inside root structures, while inhibiting the transfer of cations to the leaf. In fact, it aids in the formation of a physical barrier surrounding the roots, known as a rhizosheath, and contributes to the synthesis of ACC deaminase, an enzyme that minimizes the suppression of growth mediated by ethylene. These sets of reactions occur to limit the effects of abiotic stress, such as salt stress, so that ethylene accumulation is controlled in the plant, root shape/architecture is modified, hydraulic conductivity is improved, and the hormone status becomes more balanced (Mousavi and Karami, 2022). These modifications in the roots can help plants to absorb greater amounts of nutrients and to have more soil moisture available. PGPB can induce genes that encode proteins involved in cellular proliferation and metabolism, especially amino-acid metabolic processes and actions in the tricarboxylic-acid cycle (Etesami and Beattie, 2018; Mousavi and Karami, 2022). More tolerance in some accessions in the present study and better biochamical and physiological performance can be attributed to these mechanisms.

The decrease in plant growth, under NaCl exposure, can be attributed to the changes in osmotic pressure by salinity, as a result of excessive ion accumulation in plants, followed by nutritional disequilibrium (Munns, 2002; Olmo et al., 2019). Leaf area influences photosynthetic performance and the buildup of photosynthetic products. In agreement with the present results on licorice accessions, previous research showed that salinity reduced leaf area expansion in calotropis (*Calotropis procera*) (Melo et al., 2021) and potato (*Solanum tuberosum* L.) (Chourasia et al., 2021). Salinity caused stomatal limitation and led to lower water content in plants. Similar to the current results, a lower level of RWC was observed in tobacco plants because of salt stress (Khatri and Rathore, 2019).

In many crops, *Azotobacter* is known to have a considerable effect on plant water status, with a mitigating role in response to salt stress, such as the case in purple basil (*Ocimum basilicum* L.) (Salimi et al., 2021) and wheat (*Triticum aestivum* L.) (El-Nahrawy and Yassin, 2020). *Azotobacter* may increase stomatal conductivity by preventing damage to the stomatal aperture. A higher level of WUE under a combined treatment of salinity and *Azotobacter* inoculation reportedly resulted in moisture conservation in stressed plants as they accumulated higher amounts of organic solutes (Khatri and Rathore, 2019).

Fv/Fm is a sensitive measure of photosynthetic activity in plants (Vishnupradeep et al., 2022). Lower values of Fv/Fm reportedly occur in stressed plants, which is parallel to a decrease in quenching ability by photochemicals within the PSII. Salt stress has been observed to reduce the maximal quantum yield (Fv/Fm) in strawberry plants (Avestan et al., 2021) and common purslane (*Portulaca oleracea* L.) (Hnilickova et al., 2021), which confirms the findings of the present research on licorice (Qu et al., 2012). Salt stress also lowered the conversion efficiency of photosynthetic light energy and impeded the photosynthetic electron transfer rate and actual photochemical efficiency of PSII, according to relevant research on the issue (Cha-Um and Kirdmanee, 2009). In partial agreement with the present results, previous research on *Atriplex centralasiatica* Iljin showed that Fv/Fm values were substantially reduced by exposing the plants to 300 mM NaCl (Qiu et al., 2003). Under high levels of salinity stress, the decrease in Fv/Fm reportedly hampered light-harvesting reactions (Qiu et al., 2003; Yan et al., 2015).

Salt stress caused an increase in membrane leakage due to enhanced levels of peroxidation in membrane lipids, resulting in the loss of membrane integrity (Ahmad et al., 2015). Salinity caused membrane lipid peroxidation, thereby causing enhanced levels of membrane permeability and a higher MDA content (Behdad et al., 2020). While NaCl molecules are ionized, there is a parallel increase in the Cl^-^ concentration within cell membranes, which reduces the pH of the cell membrane and breaks down hydrogen bonding inside its protein molecules when exposed to salinity. As a result, membrane integrity may be compromised, resulting in further ion leakage and a higher level of cellular permeability (Çoban and Baydar, 2016). A previous report indicated that root-associated microorganisms can minimize membrane lipid peroxidation by improving free radical scavenging mechanisms while providing membrane robustness against abiotic stress (Haghighi et al., 2022). An increase in MSI can result from cellular and structural functions that are assisted by *Azotobacter*, as they help to prevent salinity stress from damaging the structural and functional deterioration of cell membranes (Kuhla et al., 2021). Similar to the current research, the increase in MSI by *Azotobacter* was reportedly observed in previous cases of study on soybean (*Glycine max* L. Merrill) (Yaghoubian et al., 2021), rice (*Oryza sativa*) (Kumar et al., 2021) and faba bean (*Vicia faba* L.) (El-Flaah et al., 2021).

Changes in stomatal size and density can occur as a result of genetic factors and/or plant development in response to a variety of environmental factors (Bertolino et al., 2019). The exposure of plants to salinity usually leads to close stomata, which corresponds with more efficient water conservation. Meanwhile, trichomes are thorn-like outgrowths on aerial parts of plants. In particular, salinity stress increases trichome density. The increase in trichome numbers and length means that more areas of the plant surface become physically covered by the trichomes for more efficient water conservation to relieve abiotic stress (Mousavi et al., 2022). It was observed that trichome density increased in response to salt stress in buffel grass (*Cenchrus ciliaris* L.) (Wasim et al., 2022), catnip (*Nepeta cataria* L.) (Lungoci et al., 2022), and smooth flatsedge (*Cyperus laevigatus* L.) (Mumtaz et al., 2021), which confirms the relevant results in the present study. It is now widely assumed that halotolerant PGPB has an inherent potential to assist in coping with high-saline conditions and to facilitate plant growth through different direct/indirect mechanisms that benefit plants (Sagar et al., 2022).

## 5 Conclusions

Salinity stress usually causes high costs in agricultural production. Its destructive effects limit plant development, resulting in lower amounts of food production. Plants have an inherent ability to respond to specific types of stress, but the application of PGPB can stimulate crop growth through direct and indirect mechanisms, despite salt-induced stress situations. This can be perceived as an emerging strategy for increasing the current levels of food production. Inoculating plants with PGPB can make them more resistant to salinity. PGPB strains can boost growth-related parameters and enhance plant productivity under salinity stress. PGPB has a vital role in enhancing plant growth and crop productivity, maintaining balanced nutrient cycling, controlling pesticide pollution, and increasing the production of secondary metabolites. The recent focus on developing a combination of formulations for mitigating the effects of salt stress and increasing the tolerance of plants can promote sustainable agriculture. The use of advanced molecular techniques to search for halotolerant PGPB can serve as a strategy to assist in agricultural endeavors in salt-affected soils. Also, this approach can facilitate the exploration of biochemical mechanisms, genes, and signaling pathways that help to neutralize salinity stress in crops, thereby highlighting the role of halotolerant PGPB in improving crop growth under salinity stress.

## Data availability statement

The original contributions presented in the study are included in the article/**Supplementary Material**. Further inquiries can be directed to the corresponding author.

## Author contributions

SM Investigation, Methodology, Writing-Original draft preparation. AK Supervision, Validation, Reviewing and Editing and Funding. FM Validation, Data Analysis, Reviewing and Editing. All authors contributed to the article and approved the submitted version.

## Funding

The authors wish to extend they’re thanks to Shiraz University for their financial support.

## Acknowledgments

The authors wish to extend their thanks and appreciation to Shiraz University for their support.

## Conflict of interest

The authors declare that the research was conducted in the absence of any commercial or financial relationships that could be construed as a potential conflict of interest.

## Publisher’s note

All claims expressed in this article are solely those of the authors and do not necessarily represent those of their affiliated organizations, or those of the publisher, the editors and the reviewers. Any product that may be evaluated in this article, or claim that may be made by its manufacturer, is not guaranteed or endorsed by the publisher.
