# Supplementary material for: Photosynthesis and chlorophyll fluorescence of Iranian licorice (Glycyrrhiza glabra l.) accessions under salinity stress
Source: Front Plant Sci. 2022 Oct 7;13:984944. doi: 10.3389/fpls.2022.984944 (PMC9585319; doi:10.3389/fpls.2022.984944)
Supplement: Supplementary file 1 [file DataSheet_1.docx]

Table S1. Analysis of variance of Irainin licorice accessions’ morphophysiological traits under studied treatments.

| Source | RWC | | | WUE | | | Electrolyte leakage | | | MSI | | | MDA | | |
| --- | --- | --- | --- | --- | --- | --- | --- | --- | --- | --- | --- | --- | --- | --- | --- |
|  | df | F | P | df | F | P | df | F | P | df | F | P | df | F | P |
| Accession | 15 | 1.64 | 0.07 | 15 | 5.12 | 0.00 | 15 | 2.36 | 0.00 | 15 | 6.05 | 0.00 | 15 | 4.06 | 0.00 |
| Bacterial inoculation | 1 | 58.51 | 0.00 | 1 | 5.62 | 0.01 | 1 | 1.23 | 0.27 | 1 | 5.92 | 0.01 | 1 | 21.22 | 0.00 |
| Salinity | 1 | 87.51 | 0.00 | 1 | 35.55 | 0.00 | 1 | 92.28 | 0.00 | 1 | 60.21 | 0.00 | 1 | 130.32 | 0.00 |
| Accession×Bacterial inoculation | 15 | 1.39 | 0.16 | 15 | 0.52 | 0.92 | 15 | 1.16 | 0.31 | 15 | 1.46 | 0.13 | 15 | 1.64 | 0.07 |
| Accession×Salinity | 15 | 1.74 | 0.05 | 15 | 2.05 | 0.01 | 15 | 1.89 | 0.03 | 15 | 2.19 | 0.01 | 15 | 1.83 | 0.03 |
| Bacterial inoculation×Salinity | 1 | 0.59 | 0.44 | 1 | 0.41 | 0.52 | 1 | 0.15 | 0.69 | 1 | 0.00 | 0.95 | 1 | 0.62 | 0.43 |
| Accession×Bacterial inoculation×Salinity | 15 | 0.49 | 0.94 | 15 | 0.52 | 0.92 | 15 | 0.94 | 0.52 | 15 | 1.24 | 0.25 | 15 | 0.74 | 0.73 |
| Error | 128 | - | - | 128 | - | - | 128 | - | - | 128 | - | - | 128 | - | - |

Table S2. Analysis of variance of Irainin licorice accessions’ morphophysiological traits under studied treatments.

| Source | Chlorophyll a | | | Chlorophyll b | | | Total Chlorophyll | | | Carotenoids | | | F0 | | | Fv/Fm | | | |
| --- | --- | --- | --- | --- | --- | --- | --- | --- | --- | --- | --- | --- | --- | --- | --- | --- | --- | --- | --- |
|  | df | F | P | df | F | P | df | F | P | df | F | P | df | F | P | df | F | P |  |
| Accession | 15 | 2.45 | 0.00 | 15 | 2.51 | 0.00 | 15 | 1.69 | 0.06 | 15 | 1.49 | 0.11 | 15 | 2.57 | 0.00 | 15 | 7.44 | 0.00 |  |
| Bacterial inoculation | 1 | 134.45 | 0.00 | 1 | 56.59 | 0.00 | 1 | 64.90 | 0.00 | 1 | 12.83 | 0.00 | 1 | 30.10 | 0.00 | 1 | 32.44 | 0.00 |  |
| Salinity | 1 | 224.61 | 0.00 | 1 | 93.8 | 0.00 | 1 | 104.60 | 0.00 | 1 | 44.57 | 0.00 | 1 | 11.69 | 0.00 | 1 | 5.86 | 0.01 |  |
| Accession×Bacterial inoculation | 15 | 0.98 | 0.48 | 15 | 1.85 | 0.03 | 15 | 1.25 | 0.71 | 15 | 0.35 | 0.98 | 15 | 0.89 | 0.57 | 15 | 0.77 | 0.71 |  |
| Accession×Salinity | 15 | 1.64 | 0.07 | 15 | 1.25 | 0.24 | 15 | 1.72 | 0.99 | 15 | 0.37 | 0.98 | 15 | 0.45 | 0.96 | 15 | 0.28 | 0.99 |  |
| Bacterial inoculation×Salinity | 1 | 4.27 | 0.04 | 1 | 2.1 | 0.15 | 1 | 0.28 | 0.54 | 1 | 0.05 | 0.81 | 1 | 0.04 | 0.84 | 1 | 0.37 | 0.54 |  |
| Accession×Bacterial inoculation×Salinity | 15 | 1..01 | 0.44 | 15 | 1.66 | 0.06 | 15 | 1.17 | 1.00 | 15 | 0.24 | 0.99 | 15 | 0.33 | 0.99 | 15 | 0.17 | 1.00 |  |
| Error | 128 | - | - | 128 | - | - | 128 | - | - | 128 | - | - | 128 | - | - | 128 | - | - |  |

Table S3. Analysis of variance of Irainin licorice accessions’ morphophysiological traits under studied treatments.

| Source | Fm | | | Fv | | | Sub-Stomatal CO_2_ | | | Stomatal Conductance of CO_2_ | | | Photosynthetic rate | | | Leaf area | | | |
| --- | --- | --- | --- | --- | --- | --- | --- | --- | --- | --- | --- | --- | --- | --- | --- | --- | --- | --- | --- |
|  | df | F | P | df | F | P | df | F | P | df | F | P | df | F | P | df | F | P |  |
| Accession | 15 | 9.62 | 0.00 | 15 | 9.73 | 0.00 | 15 | 15.49 | 0.00 | 15 | 13.40 | 0.00 | 15 | 8.31 | 0.00 | 15 | 3.04 | 0.00 |  |
| Bacterial inoculation | 1 | 31.95 | 0.00 | 1 | 34.39 | 0.00 | 1 | 55.06 | 0.00 | 1 | 11.11 | 0.00 | 1 | 77.60 | 0.00 | 1 | 46.20 | 0.00 |  |
| Salinity | 1 | 7.79 | 0.00 | 1 | 8.18 | 0.00 | 1 | 186.3 | 0.00 | 1 | 98.23 | 0.00 | 1 | 237.08 | 0.00 | 1 | 103.80 | 0.00 |  |
| Accession×Bacterial inoculation | 15 | 0.94 | 0.52 | 15 | 0.83 | 0.64 | 15 | 0.76 | 0.71 | 15 | 3.73 | 0.00 | 15 | 1.79 | 0.02 | 15 | 1.00 | 0.45 |  |
| Accession×Salinity | 15 | 0.16 | 1.00 | 15 | 0.12 | 1.00 | 15 | 1.23 | 0.25 | 15 | 5.32 | 0.00 | 15 | 2.72 | 0.00 | 15 | 0.99 | 0.47 |  |
| Bacterial inoculation×Salinity | 1 | 0.09 | 0.76 | 1 | 0.32 | 0.57 | 1 | 0.83 | 0.36 | 1 | 0.66 | 0.42 | 1 | 1.32 | 0.25 | 1 | 3.88 | 0.05 |  |
| Accession×Bacterial inoculation×Salinity | 15 | 0.37 | 0.98 | 15 | 0.33 | 0.99 | 15 | 1.88 | 0.03 | 15 | 3.75 | 0.00 | 15 | 2.12 | 0.01 | 15 | 0.59 | 0.87 |  |
| Error | 128 | - | - | 128 | - | - | 128 | - | - | 128 | - | - | 128 | - | - | 128 | - | - |  |

Table S4. Analysis of variance of Irainin licorice accessions’ morphophysiological traits under studied treatments.

| Source | Transpiration rate | | | Carboxylation efficiency | | | Number of stomatal | | | Number of trichomes | | | Stomata length | | | Stomata width | | | |
| --- | --- | --- | --- | --- | --- | --- | --- | --- | --- | --- | --- | --- | --- | --- | --- | --- | --- | --- | --- |
|  | df | F | P | df | F | P | df | F | P | df | F | P | df | F | P | df | F | P |  |
| Accession | 15 | 13.16 | 0.00 | 15 | 4.08 | 0.00 | 15 | 207.28 | 0.00 | 15 | 275.02 | 0.00 | 15 | 88.85 | 0.00 | 15 | 23.2 | 0.00 |  |
| Bacterial inoculation | 1 | 73.42 | 0.00 | 1 | 62.69 | 0.00 | 1 | 977.04 | 0.00 | 1 | 700.62 | 0.00 | 1 | 285.64 | 0.00 | 1 | 133.28 | 0.00 |  |
| Salinity | 1 | 143.15 | 0.00 | 1 | 119.35 | 0.00 | 1 | 3089.7 | 0.00 | 1 | 1524.7 | 0.00 | 1 | 923.35 | 0.00 | 1 | 418.35 | 0.00 |  |
| Accession×Bacterial inoculation | 15 | 0.86 | 0.60 | 15 | 0.96 | 0.50 | 15 | 18.66 | 0.00 | 15 | 25.50 | 0.00 | 15 | 6.46 | 0.00 | 15 | 5.26 | 0.00 |  |
| Accession×Salinity | 15 | 1.42 | 0.14 | 15 | 1.09 | 0.36 | 15 | 58.49 | 0.00 | 15 | 21.98 | 0.00 | 15 | 6.56 | 0.00 | 15 | 10.12 | 0.00 |  |
| Bacterial inoculation×Salinity | 1 | 5.74 | 0.01 | 1 | 4.89 | 0.02 | 1 | 125.57 | 0.00 | 1 | 102.33 | 0.00 | 1 | 18.52 | 0.00 | 1 | 0.39 | 0.53 |  |
| Accession×Bacterial inoculation×Salinity | 15 | 1.47 | 0.12 | 15 | 1.46 | 0.13 | 15 | 32.89 | 0.00 | 15 | 16.52 | 0.00 | 15 | 7.02 | 0.00 | 15 | 2.67 | 0.00 |  |
| Error | 128 | - | - | 128 | - | - | 128 | - | - | 128 | - | - | 128 | - | - | 128 | - | - |  |

Figure S1. Chlorophyll a/b ratio in studied accessions of Iranian licorice. According to the analysis of variance, the above present mean comparison is the only effect that showed a significant difference. Mean values with the same letters are not significantly different (p ≤ 0.05), Tukey test. Bars stand for standard error (SE).

Figure S2. Chlorophyll a/b ratio under bacterial treatment in Iranian licorice accessions. According to the analysis of variance, the above present mean comparison is the only effect that showed a significant difference. Mean values with the same letters are not significantly different (p ≤ 0.05), Tukey test. Bars stand for standard error (SE).

Figure S3. Chlorophyll a/b ratio under salinity stress in Iranian licorice accessions. According to the analysis of variance, the above present mean comparison is the only effect that showed a significant difference. Mean values with the same letters are not significantly different (p ≤ 0.05), Tukey test. Bars stand for standard error (SE).

Figure S4. Total Chlorophyll/Carotenoid ratio under integrated salinity stress and Iranian licorice accessions. According to the analysis of variance, the above present mean comparison is the only effect that showed a significant difference. Mean values with the same letters are not significantly different (p ≤ 0.05), Tukey test. Bars stand for standard error (SE).

Figure S5. Fv/Fm ratio in studied accessions of Iranian licorice. According to the analysis of variance, the above present mean comparison is the only effect that showed a significant difference. Mean values with the same letters are not significantly different (p ≤ 0.05), Tukey test. Bars stand for standard error (SE).

Figure S6. Fv/Fm ratio under bacterial treatment in accessions of Iranian licorice. According to the analysis of variance, the above present mean comparison is the only effect that showed a significant difference. Mean values with the same letters are not significantly different (p ≤ 0.05), Tukey test. Bars stand for standard error (SE).

Figure S7. Fv/Fm ratio under salinity stress in accessions of Iranian licorice. According to the analysis of variance, the above present mean comparison is the only effect that showed a significant difference. Mean values with the same letters are not significantly different (p ≤ 0.05), Tukey test. Bars stand for standard error (SE).

Figure S8. Fm/F0 ratio under salinity stress in accessions of Iranian licorice. According to the analysis of variance, the above present mean comparison is the only effect that showed a significant difference. Mean values with the same letters are not significantly different (p ≤ 0.05), Tukey test. Bars stand for standard error (SE).

Figure S9. Fv/F0 ratio under salinity stress in accessions of Iranian licorice. According to the analysis of variance, the above present mean comparison is the only effect that showed a significant difference. Mean values with the same letters are not significantly different (p ≤ 0.05), Tukey test. Bars stand for standard error (SE).
